# Development and Applications of CO_2_-Responsive Gels in CO_2_ Flooding and Geological Storage

**DOI:** 10.3390/gels9120936

**Published:** 2023-11-29

**Authors:** Yanxu Ding, Yang Zhao, Xin Wen, Yueliang Liu, Ming Feng, Zhenhua Rui

**Affiliations:** 1 National Key Laboratory of Petroleum Resources and Engineering, China University of Petroleum (Beijing), Beijing 102249, China; 2021210398@student.cup.edu.cn (Y.D.); wenxin990825@163.com (X.W.); sdliuyueliang@163.com (Y.L.); ruizh@cup.edu.cn (Z.R.); 2 College of Petroleum Engineering, China University of Petroleum (Beijing), Beijing 102249, China; 3 CNPC Engineering Technology R&D Company Limited, Beijing 102249, China; fengmingdri@cnpc.com.cn

**Keywords:** CCUS, gas channeling, CO_2_ leakage, CO_2_ responsive gel, comprehensive review

## Abstract

Gel systems are widely used as plugging materials in the oil and gas industry. Gas channeling can be mitigated by reducing the heterogeneity of the formation and the mobility ratio of CO_2_ to crude oil. Cracks and other CO_2_ leaking pathways can be plugged during the geological storage of CO_2_ to increase the storage stability. By adding CO_2_-responsive groups to the classic polymer gel’s molecular chain, CO_2_ responsive gel is able to seal and recognize CO_2_ in the formation while maintaining the superior performance of traditional polymer gel. The application of CO_2_ responsive gels in oil and gas production is still in the stage of laboratory testing on the whole. To actually achieve the commercial application of CO_2_ responsive gels in the oil and gas industry, it is imperative to thoroughly understand the CO_2_ responsive mechanisms of the various types of CO_2_ responsive gels, as well as the advantages and drawbacks of the gels and the direction of future development prospects. This work provides an overview of the research progress and response mechanisms of various types of CO_2_ responsive groups and CO_2_ responsive gels. Studies of the CO_2_ responsive gel development, injectivity, and plugging performance are comprehensively reviewed and summarized. The shortcomings of the existing CO_2_ responsive gels system are discussed and the paths for future CO_2_ responsive gel development are suggested.

## 1. Introduction

The demand for energy is growing worldwide, and the majority of the energy that people need is currently provided by fossil fuels [1]. This also leads to the release of a large amount of greenhouse gases such as CO_2_, CH_4_, N_2_O, HFCs, PFCs, and ReF_6_ into the atmosphere, causing the Earth’s temperature to rise day by day. According to the National Weather Service, the earth’s temperature has risen by 0.7 °C since the 19th century [2]. CO_2_ is one greenhouse gas and makes up more than 60% of the greenhouse effect. The global cumulative emission reduction in CCUS is 5.5 × 10^11^ to 1.017 × 10^12^ t CO_2_ to accomplish the goal of a 1.5 °C temperature rise control by the year 2100. The potential of CCUS to reduce global emissions has been assessed by the International Energy Agency and the Intergovernmental Panel on Climate Change (IPCC). To achieve near zero emissions globally by 2070, CCUS technology must accumulate an emission reduction of about 15% [3,4,5,6].

The development of CO_2_ capture utilization and storage (CCUS) as an important technology for carbon neutrality is highly promising. CO_2_-based enhanced oil recovery (CO_2_-EOR) in hydrocarbon reservoirs is one of the most economically attractive means to achieve large-scale CO_2_ utilization and geological storage. However, CO_2_ channeling through high-permeability features seriously inhibits the performance of CO_2_-EOR. In addition to gas channeling issues, in the process of CO_2_ flooding, the injected CO_2_ can also cause inorganic salt precipitation (such as metal carbonates) and asphaltene precipitation, leading to a decrease in reservoir permeability. Due to the fact that precipitation is also generated during water flooding, and the mechanism of precipitation generation is similar to CO_2_ flooding It is possible to refer to the treatment method of water flooding and introduce inhibitors into the CO_2_ flooding process to hinder or reduce the generation of precipitation. Also, there is an urgent need for CO_2_ geological storage technology to solve the problem of potential CO_2_ leakage via cracks, faults, and other high-permeability channels. Injecting CO_2_ into hydrocarbon reservoirs is an attractive way to achieve the effective utilization and storage of CO_2_ [7,8]. Current CCUS projects are mainly applied to conventional oil and gas reservoirs, in which 0.1 to 0.6 tons of crude oil can be produced for every 1 ton of CO_2_ injected. The next stage of research mainly focuses on improving the recovery rate of unconventional oil and gas reservoirs with carbon dioxide. How to effectively inject CO_2_ into shale or coal bed methane remains a key research and development direction for improving the recovery performance of such oil and gas reservoirs [9,10]. When the source and sink matching conditions are suitable, some CCUS projects in China have lower costs than the enhanced oil recovery (EOR) benefits, and have the potential for negative cost reduction [11,12]. In terms of CO_2_-enhanced oil recovery and geological storage, it is a huge challenge to choose a suitable CO_2_ sealing agent in order to control the migration of CO_2_ in the formations. As a new type of plugging agent, CO_2_-responsive gel can directionally identify and plug CO_2_ in the formation, and has good acid resistance. It has high application potential in the CO_2_ sealing of formations, and has received widespread attention from researchers [13,14].

## 2. Gas Channeling Control in CO_2_-EOR

**Figure 1:**
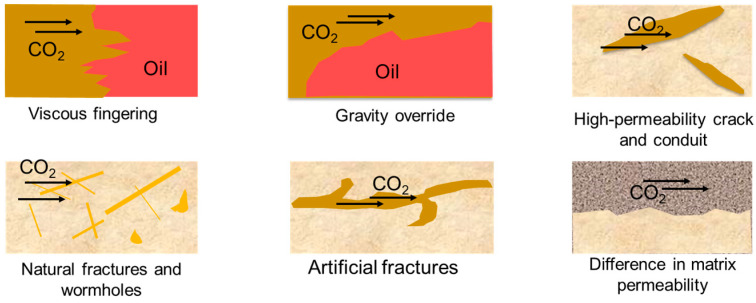
Formation mechanisms of gas channeling.

Gas channeling, as the primary issue that restricts the significant improvement of CO_2_-EOR, requires an understanding of its generation mechanisms. The factors causing gas channeling can be attributed to the significant physical differences between CO_2_ and crude oil and reservoir heterogeneities. The former can be divided into two situations (see Figure 1): (1) the gravity overriding phenomenon: during CO_2_ flooding, due to the low density of CO_2_, a large amount of CO_2_ will gradually migrate above the crude oil, ultimately forming a gas channel at the top of the crude oil seepage channel [15]; (2) the viscous fingering phenomenon: the viscosity of CO_2_ is lower than the viscosity of crude oil, which can cause uneven propulsion during the displacement process. When the flow rate of CO_2_ in a local area is too fast, gas channeling will occur [16]. The gas channeling caused by reservoir heterogeneity can be divided into the following scenarios: (1) when there are high-permeability channels such as natural fractures, artificial fractures, wormholes, and conduits, CO_2_ will bypass the matrix and cannot displace the crude oil inside [17,18]; (2) when there is a significant difference in the permeabilities of different layers or zones, CO_2_ preferentially flows through high-permeability reservoirs [19]; (3) the presence of wormholes, ducts, and high-permeability cracks in the matrix leads to the ineffective flow of CO_2_ [20].

At present, there are several methods to mitigate oil and gas channeling in CO_2_ flooding: water-alternating-gas (WAG) injection, the direct thickening of CO_2_, hydrogel plugging, foam plugging, and nanoparticle plugging. These methods for suppressing CO_2_ gas migration have been proven to be effective in practical applications and laboratory simulations. The main purpose of this section is to figure out the sealing mechanisms of different CO_2_ channeling control methods and the parameters that affect their effectiveness.

### 2.1. Water-Alternating-Gas (WAG) Injection

WAG is considered a reliable method to suppress gas channeling during CO_2_ flooding [21,22]. Awan et al. reported that, in the WAG process, CO_2_ flooding provides high sweep efficiency at the micro level, while water flooding provides high sweep efficiency at the macro level. The organic combination of the two results in a significant improvement in WAG oil recovery overall [23]. According to Leeuwenburg’s explanation, the injected water during WAG oil displacement can adjust the CO_2_ flow to a certain extent. If CO_2_ is suppressed from flowing to high-permeability zones, it is forced to displace oil left in low-permeability zones, thereby improving the overall oil recovery performance [24]. Kamali et al. also proposed a similar view, using three oil displacement methods: continuous CO_2_ injection, simultaneous CO_2_ and water injection, and WAG injection to conduct displacement experiments on sandstone cores. It was found that WAG had the best effect and continuous CO_2_ injection had the worst effect. Then, numerical simulation experiments were conducted, and it was found that the presence of injected water during the WAG process effectively reduced the permeability of CO_2_, thereby reducing the mobility ratio of CO_2_ to crude oil [25]. Han explained the enhanced oil recovery mechanism of WAG from the perspective of miscible displacement, and they believe that an increased volume of injected water significantly increases the injection pressure of CO_2_, making it easy for CO_2_ to mix with crude oil. This reduces the interfacial tension between crude oil and CO_2_, as well as the viscosity of crude oil, allowing more formation crude oil to be extracted from the ground [26].

It is worth noting that precipitation is often generated during the WAG process, which can hinder the flow of crude oil in the formation and affect the improvement of oil recovery. According to the type of precipitation, it can be divided into organic precipitation and inorganic precipitation. Organic precipitation usually refers to asphaltene precipitation [27]. Due to the injection of foreign fluids, changes in the thermodynamic parameters (temperature and pressure) and composition of the crude oil system will lead to aggregation reactions of asphaltene in the crude oil, resulting in solid asphaltene precipitation. The generated asphaltene precipitate will block the channel and seal the reservoir pores, ultimately leading to a decrease in reservoir permeability. Usually, a precipitation inhibitor is added to the injected fluid to hinder the flocculation of unstable asphaltene or make the generated asphaltene precipitate easy to wash out, thereby alleviating the blocking effect of precipitation on the formation. Inorganic precipitation mainly includes metal carbonates. During the process of alternating water and gas injection, some CO_2_ dissolves in the injected water, converting it into acidic carbonated water and accumulating CO_3_^2−^ in the aqueous solution. When water contains metal scale ions such as Mg^2+^ and Ca^2+^, CO_3_^2−^ will combine with metal scale ions. When the concentration of metal carbonate reaches a critical value, precipitation occurs. These sediments can block small pores, leading to a decrease in reservoir permeability. In addition, acidic carbonated water can also erode the reservoir, alter its permeability and pore structure, and cause metal scale ions in the reservoir to enter the injected water, further exacerbating the generation of precipitation [28]. Therefore, for the injected water in WAG, chelating agents should be used before injection into the formation to reduce the content of Ca^2+^ and Mg^2+^. Alternatively, inhibitors can be added to inhibit the formation of precipitation.

The recovery efficiency of WAG oil displacement will be greatly impacted by the injection parameters, which include porosity, permeability, and other formation properties, as well as injection parameters like the water to gas slug ratio. Hao et al. first used a thin tube experiment to determine the minimum miscibility pressure of CO_2_ and crude oil at 22.79 MPa, and then connected three different permeability cores in parallel [29]. After CO_2_ displacement, it was changed to WAG displacement, and it was found that under both displacement conditions, the core with the highest permeability contributed the most to the recovery performance, both exceeding 90%. The injection pressures were kept at 15 MPa and 25 MPa, respectively, with oil recovery efficiencies of 33.01% and 39.42%, respectively. A higher injection pressure was beneficial for oil recovery improvement [30]. Hosseini and Wang et al. found that, after CO_2_ and WAG displacement, the oil permeability and porosity of the core significantly decreased, and some areas of the core showed a reversal of wettability. They attributed this phenomenon to the presence of CO_2_-caused precipitation and the accumulation of asphaltene in the crude oil, blocking some channels, resulting in a decrease in crude oil permeability and core porosity. A combination with surfactants or other types of chemical inhibitors during WAG flooding has been reported to reduce asphaltene precipitation [31,32].

### 2.2. Direct Thickening of CO_2_

The density and viscosity of CO_2_ gas can be increased by thickening it with polymers, which reduces gas channeling issues brought on by gravity overlap and viscous fingering phenomena [33,34]. Brien believes that the increase in density and viscosity can be achieved by controlling the concentration of the added polymer [35]. Polymers enhance the density and viscosity of CO_2_ at different levels by dissolving them in CO_2_. In general, the higher the molecular weight of a polymer, the greater its viscosity, and the better its thickening effect on CO_2_. However, the higher the molecular weight of the polymer, the lower its solubility in CO_2_, which is unfavorable for CO_2_ thickening. Therefore, using low molecular weight polymers to thicken CO_2_ is also a feasible option. Siloxane polymers have been proven to be an effective CO_2_ thickener [36]. Bac tested the thickening ability of polydimethylsiloxane (PDMS) on scCO_2_ at 2500 psi and 130 °F. It was found that the viscosity of CO_2_ increased from 0.04 cp to 1.2 cp after thickening. In addition, the use of toluene results in the higher solubility of polymers under the same pressure conditions. Bac conducted CO_2_ core displacement experiments and found that, after adding polymers, the oil recovery rate increased, the gas breakthrough was delayed, and the oil recovery rate increased by 3.4–9% from its original value [37].

There is also a special polymer, which can form a three-dimensional grid structure through a cross-linking reaction between molecular chains, and the network can swell in water. This kind of polymer is called a gel [38,39,40]. Because gels have good plugging properties, they often block high-permeability channels in CO_2_-EOR, thus inhibiting gas channeling caused by formation heterogeneity [41]. There are two solutions for polymer gels used for CO_2_ consistency control. The first solution is in situ gel plugging, which injects the solution composed of a polymer monomer, cross-linking agent, and auxiliary agent into the formation to form a gel in the formation and block the migration of CO_2_ in the high-permeability channel. The second scheme is pre-crosslinked gel plugging, which can be directly injected into the formation after the gel has been completely formed. Alternatively, it can be processed into particles and prepared with a solution, and then injected into the formation [42]. Durucan et al. carried out the core displacement experiment of supercritical CO_2_ oil displacement, injected polyacrylamide-based polymer gel into the core, and then conducted the CO_2_ displacement experiment again, and found that the permeability of CO_2_ decreased by 99% [43].

### 2.3. Foam Injection

Foam is a gas dispersion system surrounded by liquid film prepared and stabilized by a surfactant [44,45]. Surfactants are amphiphilic compounds, which means they are composed of hydrophilic heads and hydrophobic tails. They are generally divided into four types (according to the charge of the head group): non-ionic, anionic, cationic, and zwitterionic surfactants. Yan et al. found that foam has greater effective viscosity, which can alleviate gravity overlap and the viscous fingering phenomenon during CO_2_ flooding, and improve sweep efficiency during CO_2_ flooding. Foam can also control the local flow resistance of CO_2_, forcing it into the low-permeability area and displacing the crude oil [46]. Through core displacement studies, Boud and Holbrook demonstrated for the first time that foam may be used to improve oil recovery by gas flooding. Additionally, foam can be produced in reservoir rocks under both miscible and immiscible circumstances using this water-soluble foaming agent. Ren et al. tested the effects of three different types of surfactants on CO_2_ flooding. The first two surfactants were 2 EH-PO_5_-EO_15_ and 2 EH-PO_5_-EO_9_, both of which were nonionic surfactants. The third type was the water-soluble anionic surfactant CD-1045. The phase behavior experiments conducted showed that none of these three surfactants could significantly reduce the interfacial tension between water and crude oil. However, all of them can significantly improve crude oil recovery. Compared with pure CO_2_ flooding, the three surfactants can increase oil recovery by 71%, 92%, and 54%, respectively. Moreover, the effect of improving the oil recovery is closely related to the injection scheme [47]. Zhang et al. used UC_11_AMPM, SDS, and their mixture as foaming agents, respectively, to prepare CO_2_ foams, and tested the effect of temperature on the stability of these three foams. With the increase in temperature, the stability of the foams decreased, and the foam produced by the UC_11_AMPM and SDS mixture had the best temperature resistance [48]. Combining multiple surfactants can achieve better oil recovery effects, but the proportion of different surfactants will have a significant impact on the oil displacement effect. Attarhamed and zoveidavianpoor found that the foaming performance of the mixture of AOS and TX-100 in aqueous CO_2_ foam was improved compared with that of AOS and TX-100 alone [49]. Memon et al. used AOS, TX-100, and a third surfactant, rose amidopropylamine oxide (LMDO), to control the fluidity of CO_2_ and improve oil recovery. After the water flooding of Berea sandstone using different combinations of CO_2_ and surfactant solutions at 1400 psi and 96 °C, surfactant alternating gas (SAG) injection was performed. According to core oil displacement experiments, CO_2_-SAG based on (0.6 wt% AOS + 0.6 wt% TX-100) achieved the highest recovery rate [50].

There are three main options for introducing surfactants into oil recovery processes. First, CO_2_ foam is generated from the outside and then injected into the porous medium. Secondly, the surfactant solution and CO_2_ can be injected together at the same time to form foam in porous media. Thirdly, carbon dioxide and surfactant solutions can be alternately injected, known as SAG injection. The advantages of surfactants mainly lie in reducing viscosity fingering, gravity segregation, and early CO_2_ breakthrough by changing the magnitude of viscosity and gravity. In addition to fixing CO_2_, surfactants also tend to reduce the IFT between reservoir fluids, reduce capillary forces, and thus improve crude oil recovery. The synergistic effect of multiple surfactants may produce a better profile control effect than a single surfactant, and this profile control effect is closely related to the proportion of different types of surfactants, which will also an important development direction of foam profile control and flooding in the future.

### 2.4. Nanoparticle Injection

An NP (nanoparticle) is defined as a material composed of particles with sizes between 1 nm and 100 nm [51,52]. In terms of CO_2_-EOR, nanoparticles enhance oil recovery through two pathways: improving the mobility ratio of CO_2_ to crude oil and reducing asphaltene precipitation during CO_2_ flooding [53,54]. Lu et al. designed a CO_2_ core displacement experiment and injected Al_2_O_3_ nanoparticles into the core. They found that they adsorbed asphaltene in a solution prepared from toluene and dissolved asphaltene, which means that these NPs can be used to suppress the deposition of asphaltene during CO_2_ injection in porous media. A concentration of 0.5 wt% nanoparticles and a volume ratio of 0.1 nanofluid slugs to CO_2_ slugs are considered the best conditions for inhibiting asphaltene damage during CO_2_ flooding. Compared to the cyclic injection mode, continuous CO_2_ and nanofluid injection may be more effective. The higher the mass fraction of Al_2_O_3_ nanoparticles, the lower the strength of asphaltene precipitation and the greater the decrease in interfacial tension [55]. Other studies have also reached the same conclusion that nanoparticles can reduce the interfacial tension between crude oil and CO_2_ and reduce asphaltene precipitation in crude oil [56,57]. Ehsan et al. simulated the viscosity increasing effect of Al_2_O_3_ nanoparticles with particle diameters of 1 nm, 2 nm, and 3 nm on scCO_2_ in an environment of 380 K and 20 MPa. Particles with a diameter of 1 nm have the weakest effect on CO_2_ viscosity, resulting in a 3.67-fold increase in CO_2_ viscosity. The author also compared the viscosity increasing effect of spherical Al_2_O_3_ nanoparticles and columnar CuO nanoparticles on scCO_2_, and found that the viscosity increasing effect of CuO was 3.4 times lower than that of Al_2_O_3_ [58].

**Figure 2:**
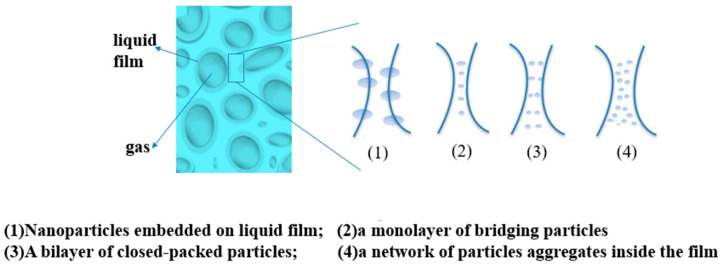
Mechanism of nanoparticles enhancing the stability of foam.

Because the surfactant is easy to be adsorbed on the rock surface and decomposes itself, the stability of foam in the formation is poor, and it is not suitable for large-scale application. Nanoparticles can effectively improve the stability of foam in the formation, which has attracted the attention of researchers. At present, there are several views on the mechanism of nanoparticles improving the strength of foam (see Figure 2): (1) nanoparticles will gather at the node intersection of the foam liquid film, hinder the liquid flow between liquid films, reduce the water loss rate of the foam liquid film, and thus improve the stability of the foam liquid film; (2) nanoparticles will form a single layer, double layer, and network of bridging particles between foam liquid films to hinder the coalescence and water loss of the foam, thus improving the stability of the foam [59,60]. Among them, the network aggregation of nanoparticles has the strongest stabilizing effect on foam. AttarHamed et al. investigated the effect of the diameter and concentration of SiO_2_ nanoparticles on the anionic surfactant effect of α-AOS-CO_2_ foam stability. The concentrations of the nanoparticles were 0.1 wt%, 0.3 wt%, 0.5 wt%, and 1 wt%, respectively. The diameters of the nanoparticles were 15 nm, 70 nm, and 250 nm. The final experimental results are shown in the figure. When the particle concentration was low, the larger the particle diameter, the better the stability effect of the foam [61]. Bayat et al. compared the stabilization effect of TiO_2,_ CuO, Al_2_O_3_, and SiO_2_ nanoparticles on CO_2_ foam. When the concentration of nanoparticles was 0.008 wt%, the stabilization effect was the best. When using SiO_2_ nanoparticles under the same conditions, the maximum increase in crude oil recovery was 17.4%. The main reason for the poor stability of nanoparticles in foam is that nanoparticles are easily adsorbed on the rock surface and agglomerated. Therefore, the better the dispersity of particles in the system, the better the stability of the foam [62,63,64].

The above four methods for preventing and controlling oil and gas migration in CO_2_ flooding have their own advantages and disadvantages, as summarized in Table 1. In order to further solve the problem of gas migration during CO_2_ flooding, in addition to making up for the shortcomings of existing technologies, efforts should also be made to develop new CO_2_-enhanced oil recovery technologies.

## 3. Pathways of CO_2_ Leakage

**Figure 3:**
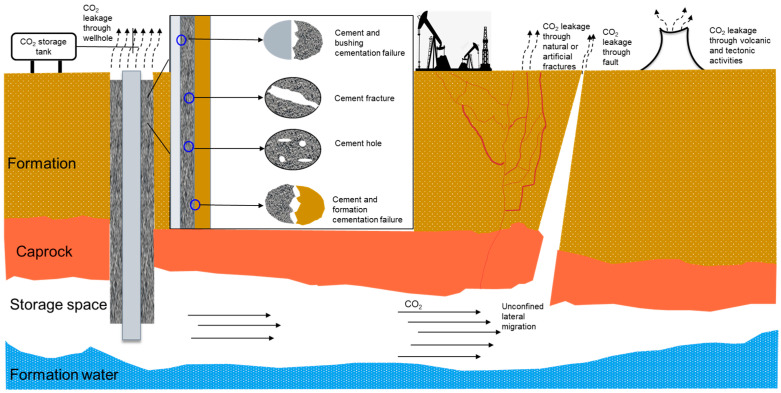
The pathways of CO_2_ leakage.

CO_2_ geological storage is the process of trapping CO_2_ emitted during the burning of fossil fuels before it enters the atmosphere, moving it via pipelines to the burial location, and then sealing it in a supercritical state in the formation (which typically includes deep salt water layers, abandoned gas layers, and abandoned oil and gas reservoirs). CO_2_ leakage is the term used to describe the phenomenon in which stored CO_2_ migrates to the ground along faults, wellbore fractures, and other formation fractures, or where it re-enters the atmosphere due to geological events (such as earthquakes or volcanic eruptions) or human activity, ultimately leading to the failure of storage (see Figure 3).

### 3.1. Potential Inflence of CO_2_ Leakage

The hazards caused by CO_2_ leakage can be roughly divided into two categories: global risks and regional risks. Global risk refers to the change in the global climate caused by CO_2_ leakage, while local risk refers to the damage to the local ecological environment caused by CO_2_ leakage [65].

After CO_2_ leakage, it will enter the atmosphere again and cause a secondary greenhouse effect [66]. Patil et al. believe that after CO_2_ leakage, the oxygen content in the soil will be significantly reduced, leading to a decrease in crop yields such as grass and soybeans. In the experiment they designed, when the CO_2_ flow rate reached 1 L/min, the yield of soybeans decreased by half [67]. The United States Department of Energy designed the “Frio Brine Pioneer Experiment”. In the experiment, the researchers injected 1600 t carbon dioxide into the sandstone layer 1550 m deep underground in an oil field northeast of Houston, Texas. Carbon dioxide caused the pH value of the brine in the storage formation to drop from nearly neutral 6.5 to 3.0, and dissolved a large number of carbonate rocks, leading to CO_2_ leakage and the pollution of drinking water. When the concentration of CO_2_ is too high, it can cause suffocation. When CO_2_ is excessively injected into the formation, it can cause excessive injection pressure to cause fractures in the formation, and, additionally, it may trigger earthquakes.

### 3.2. The Pathways of CO_2_ Leakage

There are two ways in which CO_2_ might leak. The first is known as engineering leakage and is brought on by either artificially created reservoir damage or a poorly wellbore integrity. When CO_2_ enters the formation and leaks due to natural geological activities or geological characteristics during the migration process, this leakage pathway is called natural leakage [68].

#### 3.2.1. Engineering Leakage Pathways

When the integrity of an oil well is poor, such as when there are some small cracks on the wellbore wall or near the wellbore, CO_2_ will leak along these cracks [69]. When the depth of the well is low, the injected CO_2_ cannot reach the designated storage location. The low density of CO_2_ will continuously migrate above the formation, ultimately leading to leakage. CO_2_ leakage can be avoided by restoring wellbore integrity, but this is only economically feasible when the number of wellbore repairs is small. It is not economically feasible to prevent CO_2_ leakage by repairing poorly sealed wells. Excessive CO_2_ injection pressure can lead to formation fractures and become a potential pathway for CO_2_ leakage. In addition, other human activities such as oil and gas engineering operations such as crude oil extraction and exploration can also lead to the generation of formation fractures and CO_2_ leakage [70].

#### 3.2.2. Natural Leakage Pathways

Natural leakage is a CO_2_ leakage caused by geological characteristics or the geological activities of reservoirs, and is not related to human activities [71]. The mechanisms of natural CO_2_ leakage typically include the following. Firstly, the poor sealing of the cover layer, such as the presence of cracks, may lead to CO_2_ migration through the cover layer and leakage [72,73,74]. Secondly, the presence of high-permeability channels such as fractures or faults in the formation can lead to CO_2_ migration to the ground and leakage along the fractures or faults. Bentz et al. believed that these leakage channels are usually caused by geological activities such as earthquakes and tectonic movements [75]. Therefore, geological activities are also a potential factor leading to CO_2_ leakage. The deep salt water layer serves as a good CO_2_ storage site, and salt water can provide a sealing effect on CO_2_. Due to the low density of CO_2_, it will continue to migrate laterally until it bypasses the saline layer and leaks along areas with poor sealing of the formation.

### 3.3. CO_2_-Sealing Agents

Manceau et al. suggested that existing CO_2_ plugging materials can prevent CO_2_ leakage using the following three techniques: (1) mitigating CO_2_ leakage by restoring wellbore integrity; (2) obstructing the high-speed migration of CO_2_ in cracks or faults; (3) improving the sealing of the cover layer to CO_2_ [68]. Wu et al. classified six types of sealants used to alleviate cement-related wellbore leakage. They are cementitious materials, temperature-activated resins, nano-strengthening sealants, gels, geopolymer cements, and low melting point alloys [66]. Based on the existing literature, this article summarizes the types, characteristics, functional mechanisms, and scope of applications of seven CO_2_-sealing materials, and points out the advantages and disadvantages of each material systems. The seven materials are Portland cement, geopolymer cement, resin, gel, biofilm barrier, nanoparticles, and foam, as summarized in Table 2.

## 4. CO_2_ Responsive Gel

CO_2_-responsive intelligent gel refers to a kind of gel the structure and properties of which can undergo particular transformation when contacting CO_2_. The stimulus source of this type of gel is CO_2_, which can avoid the accumulation of a large number of impurities in the response process using traditional stimulus sources. By utilizing the intelligent CO_2_ response characteristics, it can be applied to sealing CO_2_ during CO_2_ flooding and geological storage [13].

### 4.1. CO_2_ Responsive Functional Group

**Figure 4:**
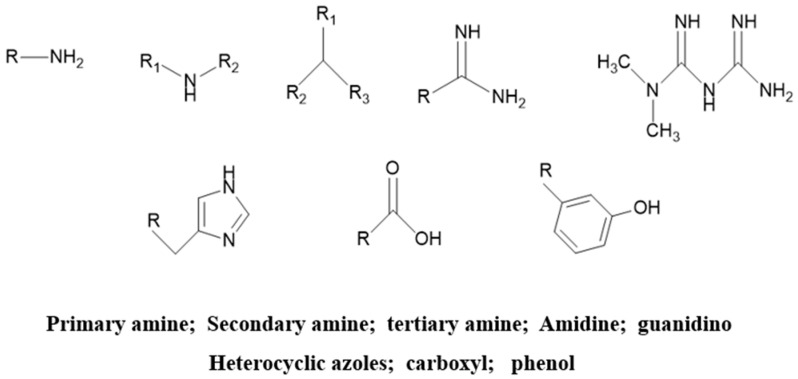
CO_2_ responsive groups.

The process of synthesizing CO_2_ responsive gels involves adding functional groups that are responsive to CO_2_ to the conventional gel’s molecular chain. As summarized in Figure 4, there are several known CO_2_ responsive functional groups: primary amine groups, amidine groups, guanidine groups, tertiary amine groups, azole-containing heterocyclic groups, carboxylic acid [89].

Primary amine groups: CO_2_ can react with primary amines at room temperature to generate negatively charged carbamate salts, and some of them are positively charged through protonation, forming bicarbonate between them, thus achieving the salt bridge bonding of primary amine groups, which is the basis for primary amine groups having CO_2_ responsiveness.

Amidine groups: Slightly stronger in alkalinity, they will be hydrolyzed to a certain extent in aqueous solution, resulting in deviations when calculating the degree of protonation of the amidine group from the amount of CO_2_ injected. When amidine group encounters CO_2_, it will generate protonated amidine salt compounds. After CO_2_ is discharged, amidine salt will appear deprotonated and return to the original state again.

Guanidine: Guanidine is the most basic CO_2_-responsive functional group, and the addition of aromatic substituents slows down alkalinity. Guanidine containing N-H bonds can form carbamates or forms of salts other than bicarbonate. The alkalinity of the guanidine group is the highest among several functional groups at present, so it has the strongest CO_2_ responsiveness, which also leads to more energy consumption during its deprotonation process. At the same time, the higher temperature required to convert bicarbonate or guanidine formate into neutral guanidine is an advantage for applications that require high temperatures.

Tertiary amino groups: They are common weakly alkaline molecular groups that are found in many polymers. Tertiary amino groups can undergo changes with CO_2_ to form cationic tertiary amine salts. When the CO_2_ concentration decreases, a reversible deprotonation reaction will occur, and the goal of a CO_2_ reversible response will be achieved finally.

Azazole-containing heterocycles: Numerous organisms include heterocyclic compounds including azole, and the molecular fragments of these compounds are present in peptides, genetic elements, and amino acids. In addition, azazole heterocyclic compounds can act as functional groups in response to CO_2_ and exhibit weak alkalinity. This polymer can produce protonated histamine salts in response to CO_2_ in solution. Imidazole functional groups can be thought of as a novel class of CO_2_-adsorbent materials since they are more stable following protonation than amidine and tertiary amine groups.

Carboxylic acid and phenols: These groups are present in the solution as anions when CO_2_ is not present. The pH of the solution steadily drops as the CO_2_ level rises until it reaches a critical value (corresponding to the solution pKa), and the functional groups progressively become neutral, which indicates a steady decline in the polymer’s solubility. Typically, these groups are added to the solution as anions or acids. In cases when the neutral form’s solubility in aqueous solution is restricted, the critical point could be noticeably higher than the pKa.

The advantages and disadvantages of each responsive group are summarized in Table 3. Based on the characteristics of each responsive group, its corresponding polymer or itself usually has the following advantages and disadvantages for CO_2_ flooding profile control and CO_2_ geological storage.

### 4.2. CO_2_ Responsivee Gels

The studies of CO_2_ responsive gels are summarized in Table 4. As early as 2003, Carretti et al. synthesized a class of polyacrylamide-based polymers (PAAm), which contained fatty alcohols and 1-methyl-2-pyrrolidone in organic solvents with a large number of primary amine groups on their side chains at room temperature. After the continuous introduction of CO_2_ into the system, the system gradually changes from the initial colloidal state to a gel, and gels can absorb a high concentration of CO_2_. The primary amino group has obvious advantages in the synthesis of CO_2_-responsive gels, but the primary amino gel is relatively slow in its response to CO_2_ [90]. In 2005, Darabi et al. found that amidine groups are responsive to CO_2_, which inspired a series of amidine gel to be designed and synthesized [91]. In 2011, Yan et al. successfully prepared CO_2_-responsive and breathable polymer vesicles using amphiphilic block copolymers containing amidine groups for the first time. They first synthesized the functional monomer N-guanidododecylacrylamide containing an amidine group, and polymerized it with polyethylene glycol macromolecules through atom transfer radical polymerization to synthesize polyethylene glycolb poly (N-guanidododecylacrylamide) (PEO-b-PAD). After introducing CO_2_ into the system, the amidine group reacts with CO_2_ to form a protonation amidine salt structure, and the volume of the vesicles will increase significantly. When argon is continuously injected into the system, the amidine salt structure will undergo deprotonation, and then return to the solid state [92].

The synthesis of amidine groups is relatively complex, and the degree of their protonation is reduced due to their easy hydrolysis in water. In 2013, Zou et al. polymerized dimethylaminoethyl methacrylate with a tertiary amino group, which was CO_2_-responsive, and with the increase in CO_2_ concentration, the degree of protonation of the polymer also increased [93]. In 2012, Hoshino et al. used 3-dimethylaminopropylacrylamide (DMAPM) containing a tertiary amine as a functional monomer to copolymerize with the thermosensitive monomer NIPAM and a crosslinking agent. A micro gel was formed in the solution. The molecular side chain of the gel contains a large number of tertiary amine groups, which can react with CO_2_, and fix CO_2_ in the gel to achieve the purpose of fixing CO_2_. This is the first successful example of using microscopic materials in solution for controllable CO_2_ absorption and release [94]. Nitrazole-containing heterocyclic compounds (usually diazoles) are widely present in organisms. Azazole heterocyclic molecular fragments can be found in amino acids, peptides, and genetic materials. At the same time, the azazole-containing heterocyclic molecules are also weakly alkaline and can respond to CO_2_ to generate protonation histamines. The protonated functional group is more stable than the previous protonated functional groups, and can be used to prepare an intelligent responsive gel for CO_2_ adsorption.

**Table 4:** CO_2_ responsive gel.

Author, Year	Responsive Functional Group	Synthesis Process	Responsive Performance of Gel	Reference
Carretti et al., 2003	Primary amine	Polymerization of allylamine to form polyacrymide.	The polymer is dissolved in the organic solvent containing fatty alcohol and 1-methyl-2-pyrrolidone, and CO_2_ gas is continuously injected, which makes the polymer change from the initial colloidal state to chemical gel.	[90]
Wang et al., 2012	Primary amine	Synthesis of a class of crosslinked porous polymer microspheres from N-methyl-N-vinylformamide (MVF) and Din-vinylformamide (DVFE).	By adjusting the ratio of MVF and DVFE monomers to control the size of polymer microspheres, the absorbable surface area inside the microspheres can be maximized. BET experiments have shown that the maximum adsorption area can reach 246 mg/g, and the total adsorption amount of CO_2_ can reach 97 mg/g.	[95]
Nagai et al., 2011	Primary amine	Regulating conventional polyaniline increases its relative molecular weight.	CO_2_ is continuously injected into the 1% polymer solution, and the solution undergoes a sol–gel–sol transition.	[96]
Suzuki et al., 2011	Primary amine	In the presence of 1,8-diazabicyclo and undecen-7-ene (DBU), a novel hydrogel was prepared by the crosslinking reaction of PAAm with CO_2_.	The CO_2_ absorption capacity of the hydrogel at room temperature is 2.8 times that of the traditional PAAm, and the absorption capacity and absorption efficiency of the polymer hydrogel are basically unchanged after multiple absorption of CO_2_.	[97]
Xu et al., 2004	Primary amine	Firstly, four urea-substituted calixarene host units were designed, and small molecule monomers with primary amine groups were obtained through selective modification.	When CO_2_ is introduced into the solution, the dimer can form linear non-covalent bond polymer through ammonium carbamate bridge bond. When competitive polar solvents are added to the solution, the hydrogen bond between the host molecule calix tetraarene is destroyed, leading to the disintegration of the supermolecule polymer.	[98]
Xiong et al., 2017	Primary amine	Mixing HMPAM and TMPDA.	After CO_2_ was introduced, the solution turned into gel, and the zero-shearing viscosity and elastic modulus increased by 360 and 400 times respectively.	[99]
Yan et al., 2023	Secondary amine	0.5 wt% NaSal and 3 wt% ENPD were dissolved in deionized water to form the initial system.	After injecting CO_2_, the viscosity of the system continues to increase and does not change after reaching a certain stage.	[100]
Yan et al., 2011	Amidine	Polyethylene glycol b-poly (N-amido dodecyl acrylamide) (PEO-b-PAD) was synthesized by aAtom transfer radical polymerization (ATRP) of N-amido dodecyl acrylamide and polyethylene glycol macromolecules. The PEO segment in this block copolymer is hydrophilic while the PAD segment is hydrophobic and can self-assemble into a vesicle structure in solution.	Alternating CO_2_/Ar injection can drive the vesicle to move towards a compound expansion/contraction movement.	[93]
Zhou et al., 2009	Amidine	The (Diphenylphosphine) ethylene terephthalate derivative containing amidine group was connected to the side group of partially azide functionalized polystyrene by Staudinger coupling method.	After dissolving the polymer in a DMF/water mixture and alternately introducing CO_2_ and N_2_, the conductivity of the system undergoes a rapid impulse change. It is proven that the polymer responds to CO_2_ gas through the protonation/deprotonation process of amidine group.	[101]
Guo et al., 2011	Amidine	A class of homopolymers containing amidine groups on its side groups were prepared by RAFT method using 4-chlorostyrene as a monomer.	Alternating the stimulation of CO_2_/N_2_ can achieve the transfer of substances in the organic/aqueous phase.	[102]
Yan et al., 2013	Amidine	A complex triblock copolymer of polyethylene glycol b poly (N-aminododecylacrylamide) b polystyrene (EAS) has been developed. Due to its amphiphilicity, the copolymer can self-assemble into micrometer-sized hollow tubular structures in aqueous solution.	After introducing CO_2_, the polymer gradually transforms from a hollow tubular body to a spherical vesicle, then into a columnar body, and finally evolves into a spherical micelle form.	[103]
Zhang et al., 2012	Amidine	Lotion polymerization of N-amido dodecyl acrylamide with styrene monomer in CO_2_ gas environment.	At 60 °C, continuous introduction of N_2_ neutral lotion molecules into the product will cause condensation; when CO_2_ gas is introduced, ionic lotion repel each other statically, leading to reversible dispersion.	[104]
Su et al., 2012	Amidine	Direct lotion polymerization of styrene monomer in 2,2-azabis (2-neneneba imidazoline) di Bicarbonate initiator.	After CO_2_ is introduced, colloidal particles between lotion repel and cause lotion dispersion.	[105]
Han et al., 2012	Tertiary amine	Copolymerization of DMAEMA with N-isopropylacrylamide (NIPAM) monomer using RAFT method to synthesize P (DMAEMA co NIPAM) random copolymer.	After CO_2_ is introduced, the solution protonation degree increases, and LCST increases from 35 °C to 60 °C.	[106]
Yan et al., 2013	Tertiary amine	Polyacrylamide and Diethylamine ethyl polymethacrylate (PDMA-b-PDEAEMA) were chimed. Copolymerize into a certain proportion of monomers containing Coumarin side groups and carry out photo crosslinking.	After introducing CO_2_, the vesicles expand and the polymer volume increases.	[107]
Han et al., 2012	Tertiary amine	Polymethacrylic acid oligopoly polyethylene glycol ester co polymethacrylic acid Dimethylamine ethyl ester b polyethylene glycol b polymethacrylic acid oligopoly polyethylene glycol ester co polymethacrylic acid Dimethylamine ethylene ester.	After CO_2_ is introduced, the gel dissociates into sol, and after Ar is introduced, the sol gradually transforms into gel.	[108]
Zhao et al., 2013	Tertiary amine	Aggregating DMAEMA.	After introducing CO_2_, the particle size increases and the solution transitions from a suspended state to a glassy state.	[109]
Lei et al., 2015	Tertiary amine	Using MBA as crosslinking agent and V-50 as initiator, copolymerize FS and DMAEMA.	After CO_2_ is introduced, gel particles expand and gel particles shrink when CO_2_ is removed.	[110]
Zhang et al., 2015	Tertiary amine	Copolymerization of pyridine and NIPAM.	After CO_2_ is introduced, gel particles expand and gel particles shrink when CO_2_ is removed.	[111]
Zhang et al., 2015	Tertiary amine	PDMAEMA-PEO-PDMAEMA triblock copolymer was synthesized using ATRF method.	After CO_2_ is introduced, the sol changes into gel, and the process is reversible.	[112]
Chen at al, 2015	Tertiary amine	DEAEMA stabilized by PEGMA was obtained through lotion polymerization.	These PDEAEMA-PEGMA micro gel collapse within 5 s after CO_2_ bubbling into solution.	[113]
Zhao at al, 2019	Tertiary amine	Mixing sodium oleate (NaOA) and the small organic counterion 2, 6, 10-trimethyl-2, 6, 10-triazaundecane (TMTAD) in a 3:1 M ratio.	After introducing CO_2_, the system transitions from an aqueous solution to a viscoelastic fluid, followed by a milky white solution.	[114]
Wu et al., 2023	Tertiary amine	Mix AM and DMAPMA, add AIPI to form a responsive polymer, and finally add PEI to form a responsive gel.	When the pH of the solution is 4.5 and the immersion time is 48 h, the mass of responsive gel can be increased by 7–18 times.	[115]
Quek et al., 2013	Heterocyclic azoles	Synthesized a class of polymers with histamine side groups using RAFT polymerization method.	The polymer can respond to CO_2_ in solution to generate protonation histamines. Because the imidazole functional group is more stable than amidine group and tertiary amine group after protonation.	[116]

## 5. Application of CO_2_-Responsive Gel in CO_2_ Gas Channeling and Leakage Mitigation

**Figure 5:**
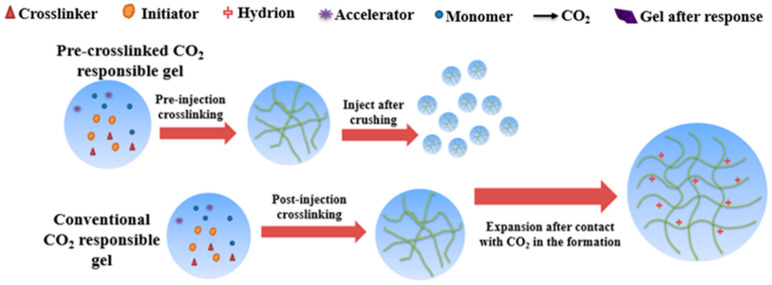
The responsive mechanism of CO_2_ responsive gel.

For the application of a CO_2_ responsive gel in CO_2_-enhanced oil recovery and geological storage, in addition to the performance of the gel itself, such as rheology, swelling, and acid resistance, etc. The environmental conditions(pressur, tempreture, etc.) in the action area of gel also greatly affect the action effect of gel Figure 5.

The key point is to observe some characteristics of gel in the actual application process, such as evaluating the plugging strength of gel by the breakthrough pressure of CO_2_ after injection of gel, or evaluating gel’s ability to adjust CO_2_ mobility by resistance coefficient and residual resistance coefficient. This paper summarizes some work on the evaluation of CO_2_ responsive gel for CO_2_ plugging ability and gas channeling inhibition ability in recent years (as shown in Table 5).

## 6. Further Development of CO_2_ Responsive Gels and Concluding Remarks

The CO_2_-responsive gel has good profile control and plugging performance, as demonstrated by numerous core-scale plugging studies. This suggests that the CO_2_ responsive gel has great application value in the field of CO_2_-EOR and CO_2_ geological storage. At present, the application of CO_2_ responsive gels on the reservoir scale is still lacking precedent, and its popularization and application in the oil and gas industry still need to solve the following problems. (1) All of the current CO_2_ responsive groups depend on H^+^ generation. In hotter conditions, CO_2_ becomes less soluble in formation water, which inhibits the generation of H^+^. The use of CO_2_ responsive gels in deep, high-temperature formation is restricted, and its sensitivity is reduced. It is necessary to explore new CO_2_ responsive materials. (2) The existing CO_2_ responsive gels are mainly used to qualitatively evaluate their CO_2_ responsiveness according to the change in their macro phase state before and after CO_2_ response. There is a lack of a systematic quantitative characterization method of gel CO_2_ responsiveness in order to screen out the ideal CO_2_ responsive gel. Moreover, CO_2_-responsive groups can be used not only in the preparation of CO_2_-responsive gels, but also in the preparation of CO_2_-responsive ionic solutions, which have broad application prospects in the field of CO_2_ capture [128,129]. (3) The current CO_2_-responsive gels are based on the mechanism of protonation of the CO_2_ responsive groups, which causes the volume expansion of the gels, and thus seal the CO_2_ migration channel of the formation. However, the protonation process of the responsive group is reversible. When the responding gel encounters a large amount of non-acid gas in the formation or the formation temperature increases, the CO_2_-responsive gel will return to the state before the response, thus losing the sealing effect on the formation. Further efforts are required to make the gels more stable and controllable. (4) In order to improve the mechanical strength, salt resistance, and temperature resistance, the CO_2_ responsive gel has been suggested to be combined with other technologies to meet the requirements of actual production in oil fields. For example, foam compound systems, organic inorganic gel compound systems, and nanoparticle compound systems.

## Figures and Tables

**Table 1:** Comparison between the different methods of CO_2_ mobility control.

Method	Advantage	Disadvantage	Influence Factor
WAG	Reduces CO_2_ loss and generate economic benefits; Increases the sweep coefficient; Relieves sticky fingering; Delays CO_2_ breakthrough; Reduces the mobility ratio; Maintains CO_2_ mixing with crude oil.	Corrodes pipelines; Unable to alleviate the phenomenon of gravitational differentiation; Initiates asphaltene precipitation; Initiates inorganic salt precipitation; Causes stress damage to the tubing; Causes water lock effect; There are many parameters involved.	Reservoir factors (temperature, pressure, thickness, porosity, permeability, saturation, heterogeneity); CO_2_ rheological properties and density; Composition and viscosity of crude oil; Water composition and mineralization; Injection parameters (injection rate, injection pressure, slug ratio); Injection scheme; Spacing between injection and production wells.
Polymers for direct thickening of CO_2_	It can be mixed with CO_2_ to form a thermodynamically stable solution; Increases CO_2_ density and viscosity; Relieves early CO_2_ breakthrough and viscous fingering.	The solubility of polymers is limited by pressure, molecular weight, and molecular chain structure, making it difficult to meet the requirements in many cases.	Reservoir factors (temperature, pressure, thickness, porosity, permeability, saturation, heterogeneity); CO_2_ rheological properties and density; Composition and viscosity of crude oil; Polymer type, molecular weight, and molecular chain structure; Injection scheme.
In situ polymer gels	Good injectability; Reduces formation heterogeneity.	Sensitive to reservoir conditions.	Reservoir factors (temperature, pressure, thickness, porosity, permeability, saturation, heterogeneity); CO_2_ rheological properties and density; Composition and viscosity of crude oil; Injection scheme; Injection parameters (injection pressure, injection rate, injection fluid concentration).
Preformed polymer gels	Reduces formation heterogeneity; Low sensitivity to reservoir.	Difficulty in injection and inability to act on deep formations; Only applicable to formations with strong heterogeneity or developed fractures.	Reservoir factors (temperature, pressure, thickness, porosity, permeability, saturation, heterogeneity); CO_2_ rheological properties and density; Composition and viscosity of crude oil; Injection scheme; Injection parameters (injection pressure, injection rate, injection fluid concentration).
Foam	Relieves sticky fingering; Relieves gravity differentiation; Relieves early breakthroughs; Reduces interfacial tension; Changes wettability; Easy to inject; Prevents and controls sedimentation.	Sensitive to temperature and pressure, prone to cracking; Material exchange with crude oil results in a decrease in stability; Adsorbs on the surface of rocks, resulting in ineffective sealing; Short life cycle; Changes the properties of crude oil.	Reservoir factors (temperature, pressure, thickness, porosity, permeability, saturation, heterogeneity); CO_2_ rheological properties and density; Composition and viscosity of crude oil; Injection scheme; Type and concentration of surfactants, molecular structure; Injection parameters (injection rate, injection pressure).
Nanoparticle	Reduces the mobility ratio; Prevents and controls asphaltene precipitation; Changes the wettability of rocks; Improves the stability and viscosity of foam; Reduces interfacial tension; Improves CO_2_ rheological properties.	Nanoparticles are prone to coalescence, blocking the roar channel, and failing; Large particle sizes can pollute the environment.	Reservoir factors (temperature, pressure, thickness, porosity, permeability, saturation, heterogeneity); CO_2_ rheological properties and density; Composition and viscosity of crude oil; Injection scheme; Nanoparticle type, particle size, hydrophilicity, concentration.

**Table 2:** Formation CO_2_ leakage prevention and sealing agent.

Plugging Agent	Composition	Working Region	Sealing Mechanism	Advantages	Disadvantages	References
Portland cement	21–67% alite; 6–18% ferrite; 0–49% belite; 1–17% aluminate.	Wellbore and near wellbore area.	Direct sealing.	Having long-term stability; low price.	Poor acid resistance; high CO_2_ emissions during production.	[76,77]
Geopolymer cement	48–56% SiO_2_; 23–35% Al_2_O_3_; 3–15% Fe_2_O_3_; 0–8% CaO; 0.2–1.4% MgO; 0.7–0.85% K_2_O; 0.2–0.5% SO_3_; 0.3–0.5% alkaline liquid/fly ash.	Wellbore and near wellbore area.	Direct sealing.	High temperature resistance, still usable at 1000 °C; strong acid resistance; the shrinkage rate is small, usually around 0.05%; the CO_2_ emission during the production process is about 20% of that of Portland cement, and the energy consumption is about 25% of that of the former.	The production steps are cumbersome; involves some highly corrosive materials, posing a threat to human life and safety; needs to go through the curing process.	[78,79]
Resin sealing system	Phenolic resin, epoxy resin, furan resin, and hardener.	Wellbore and near wellbore area.	Direct sealing.	High adhesive strength and easy to adjust viscosity; has good thermal stability and long-term stability; good acid resistance.	The preparation process is complex; high cost; difficult to control crosslinking reaction.	[80]
Gel	It is divided into organic gel systems and inorganic gel systems, represented by silicate gel.	Casing, microcracks, oil storage media.	Injection of polymer solution or gel particles to form blocky gel and block CO_2_ flow channel.	Good injection performance; controllable crosslinking time; can solve complex CO_2_ leakage problems.	The sealing effect is generally poor; poor thermal stability and acid resistance; cracks with a width exceeding 2 mm cannot be sealed.	[81,82]
Biofilm	A type of bacteria that can produce urease.	Enhances the integrity of wellbore cement.	Induction of urea hydrolysis to form calcium carbonate precipitation, blocking CO_2_.	Able to penetrate the pores of the formation and play a role in the near wellbore area; environmentally friendly; biomineralization process is controllable; good acid resistance.	High production costs; need to continuously transport nutrients; uneven distribution of precipitation; unable to block large-scale leaks.	[83,84]
Nanoparticles	Metal oxide particles or polymer particles with a diameter between 1 and 100 nm.	Reduces CO_2_ mobility; as a strengthening agent for cement, gel, and foam.	Deep reservoirs and deep salt water layers.	It can greatly improve the strength of gel, foam, and cement; does not pollute the environment; has good stability and acid resistance.	High cost; complex preparation process.	[85,86]
Foam	Aqueous solutions and foaming agents of surfactants.	Reduces the mobility of CO_2_ in high permeability channels.	Near wellbore area and deep reservoir.	The injection performance is good and can reach the deep part of the formation.	Low sealing strength and lack of long-term stability.	[87,88]

**Table 3:** Characteristics of each functional group.

CO_2_-Responsible Group	Advantage	Disadvantage
Amino groups (primary, secondary, tertiary)	The synthesis route is simple and there are many mature industrial monomers available for use; The group in aqueous solution has low alkalinity and is not easily hydrolyzed.	Slow reaction rate with CO_2_; Unable to respond to low concentration of CO_2_.
Amine and guanidine groups	Has rapid and sensitive CO_2_ responsiveness.	The group in aqueous solution has strong alkalinity and is easy to hydrolyze; Complex synthesis route.
Azazole-containing heterocycles	The salt generated after responding to CO_2_ is relatively stable; Widely present in organisms.	The working conditions are harsh and not suitable for geological environments.
Carboxyl and phenolic hydroxyl groups	Has good acid resistance; Easy to synthesize, with many mature industrial monomers available for use.	Sealing of wellbore cracks not applicable.

**Table 5:** CO_2_ responsive gel plugging CO_2_ experiment.

Author, Year	Synthetic Ingredient	Rheology	Experimental Design	Main Findings	Ref.
Mingwei Zhao, 2023	Sodium salicylate, acetone, sodium fluoride, erucic acid N,N-dimethyl-1,3-propanediamine (99%).	CO_2_ sensitivity: the relationship between the viscosity of gel at 25 °C and the amount of CO_2_ injected; rheological properties: viscosity modulus, elastic modulus, relaxation time.	CO_2_ plugging experiment and parallel core floods (80 °C, backpressure 4 MPa, 1038 mD).	The CO_2_ sealing rate reaches 97.45%, and as the permeability increases, the sealing rate decreases. When the maximum permeability is 2000 mD, the sealing rate is 95%. The sealing rate increases with injection volume. Increased the recovery factor of low permeability cores by 18.7%.	[114]
Dexiang Li, 2019	Modified polyacrylamide, methylamine, resorcinol.	Not mentioned.	CO_2_ plugging test with gel (80 °C, backpressure 10.28 MPa, 30 mD).	Water phase infiltration reduces by 85%, and the resistance coefficient during CO_2_ flow is greater than 29. The gel is easy to absorb near the injection points. The closer it is to the leakage point, the better the plugging effect will be. Gel stability declines under high temperatures.	[117]
JF Ho, 2016	Polyacrylic acid	Liquidity conforms to the power law equation.	Gel plugging CO_2_ experiment: remove calcium ions with Na_5_P_3_O_10_.	Gel can significantly increase the pressure gradient of gel’s CO_2_ retention, use Na_5_P_3_O_10_ to reduce the weakening of calcium ions on gel strength, and extend the action time of gel.	[118]
Shayan, 2017	Polyacrylic acid	It has shear dilution properties and conforms to the Herschel–Bulkley model. Under high calcium concentration, stability is weakened.	Gel plugging strength test (supercritical CO_2_, backpressure 1100 psi, 70 °C).	Using sodium phosphate as a chelating agent, increase the pressure gradient of supercritical CO_2_ interception to 70 psi/ft. The formation hypothesis of gel deposition layer is verified, and the protective effect of gel on cement integrity is confirmed.	[119]
Du et al., 2022	AM, AFAPE_2_0.	Shrinks under high temperature conditions, with a weight loss of 84.6% at 700 °C and a shear rate below 250 s^−1^. A pseudoplastic fluid, and an expansive fluid above a critical shear rate.	Plugging experiment (90 °C, backpressure 21 MPa).	The closer the particle diameter to the average pore size, the better the sealing effect. The sealing rate >90%. A significant power law relationship between resistance factor and residual resistance factor and concentration and rate of injected gel particles.	[120]
Pu W, 2021	AM, AFAPE_2_0.	Shrinks under high temperature conditions, with a weight loss of 84.6% at 700 °C and a shear rate below 250 s^−1^. A pseudoplastic fluid, and an expansive fluid above a critical shear rate.	Plugging experiment (68 °C, backpressure 3 MPa).	After the formation of gel plugging, CO_2_ injection pressure increased 68 times; the sealing rate reached 99%. After the formation of gel plugging, the CO_2_ flooding recovery rate increased by 23%.	[121]
Luo et al., 2022	N-erucamidopropyl-N,N-dimethylamine.	UC_22_AMPM solution will become power-law fluid after reacting with CO_2_.	Core flooding test (45 °C, backpressure 17 MPa).	The recovery rate of UC_2_2AMPM solution WAG increased by 8% compared to WAG.	[122]
Du et al., 2022	CO_2_-responsive gel and CO_2_-responsive wormlike micelles.	The flow state follows a power law equation.	Core flooding experiments (45 °C, backpressure 21 MPa).	The higher the matrix permeability, the poorer the system’s ability to improve oil recovery; the larger the crack width, the worse the gel plugging effect.	[123]
Du et al., 2022	CO_2_-responsive gel and CO_2_-responsive wormlike micelles.	The flow state follows a power law equation.	Core slab models (45 °C, backpressure 0.5 MPa).	Effect of alternate injection of gel system and CO_2_ slug on oil recovery single injection of gel system.	[123]
Ye et al., 2022	N. N-Dimethyloctylamide, propyl tertiary amine, Sodium p-toluenesulfonate.	Not mentioned.	Plugging and core flooding experiment (25 °C, backpressure 3 MPa).	The sealing rate for CO_2_ is 99.2%. Improved CO_2_ flooding oil recovery by 20%.	[124]
Li et al., 2016	Acrylamide, methamphetamine, Resorcinol.	Strength code after reaction with CO_2_ is H.	Sand pack experiments (90 °C, backpressure 10.28 MPa).	The CO_2_ gas drive permeability of sandstone has decreased by 93.8%. Oil recovery factor of low permeability core increased by 46.5%.	[125]
Welch et al., 2020	Methacrylate	Not mentioned.	Stability tests and Plugging experiments (confining pressure 0.5 MPa, maximum temperature 73 °C).	High salinity brine will reduce the stability of gel particles; when the ratio of monomer to surfactant is lower than 22.1, gel particles are not easy to gather and have good stability. The fluid loss rate during the initial sealing stage decreased by 93%. The seal fails after the pressure exceeds 5 MPa.	[126]
Ji et al., 2023	Sodium silicate, modified polyphenol, methena- Mine.	Gel strength code: G. After CO_2_ is injected, the viscosity decreases from 28.3 mPa·s to 20.5 mPa·s.	Plugging experiment (90 °C).	After the injection of gel, the injection pressure increased from 0.18 MPa to 0.64 MPa, the plugging ratio reached 70.3%, and the oil recovery increased by 10.4%.	[127]

## Data Availability

The article is a review paper and no new data have been established yet.
